# Newborn examination practices and congenital anomaly detection in public health facilities in Addis Ababa, Ethiopia: a qualitative exploratory study

**DOI:** 10.3389/fped.2026.1597031

**Published:** 2026-02-05

**Authors:** Merertu Temesgen Alemu, Fikre Abate, Courtney Mollenhauer, Hugh Brewster, Alex Habel, Ermias Mulatu, Sebastian Taylor, Mekonen Eshete

**Affiliations:** 1 Department of Pediatrics and Child Health, Yekatit 12 Hospital Medical College, Addis Ababa, Ethiopia; 2 Department of Plastic and Reconstructive Surgery, Yekatit 12 Hospital Medical College, Addis Ababa, Ethiopia; 3 Transforming Cleft Program Management Department, Toronto, ON, Canada; 4 Pediatrics and Child Health, Emeritus. Great Ormond Street Hospital for Children NHS Trust, London, United Kingdom; 5 Specialty and Rehabilitation Service Department, Ministry of Health Ethiopia, Addis Ababa, Ethiopia; 6 Department of Pediatrics and Child Health, Royal College of Pediatrics and Child Health, London, United Kingdom; 7 College of Health Sciences, School of Medicine, Surgical Department, Addis Ababa University, Addis Ababa, Ethiopia

**Keywords:** congenital anomalies, Ethiopia, examination practices, newborn, public hospitals

## Abstract

**Background:**

Congenital anomalies are a leading cause of infant morbidity and mortality, particularly in low-resource settings. Early detection through standardized newborn examinations is critical but remains underexplored in Ethiopia.

**Objective:**

This qualitative study systematically assesses neonatal examination practices in major public hospitals in Addis Ababa, Ethiopia, identifying gaps and proposing recommendations for standardized protocols.

**Methods:**

We conducted twelve key informant interviews from November 2024 to January 2025 with pediatricians, obstetrician-gynecologists, and midwives from four large public hospitals using purposive sampling based on role and neonatal care experience. Interviews were transcribed verbatim and analyzed thematically following Braun & Clarke's framework. Coding was performed by two researchers; discrepancies were resolved by consensus. Trustworthiness was enhanced through member checking, peer debriefing, triangulation, and an audit trail.

**Results:**

Examination practices are fragmented, with inconsistent use of structured checklists and inadequate documentation. Training gaps among clinicians and non-physician staff were evident. There is strong interest in adopting checklist-based approaches to improve anomaly detection.

**Conclusions:**

The findings highlight critical gaps in neonatal examination and documentation practices across the study sites. Developing a national checklist, comprehensive training programs, and mandatory protocols could strengthen early detection and management of congenital anomalies.

## Introduction

Congenital anomalies, or birth defects, are structural or functional abnormalities present at birth that can lead to significant health challenges, including early abortions, stillbirths, disabilities, and increased infant mortality (1). Globally, congenital disorders account for approximately 240,000 newborn deaths annually, with the majority occurring in low- and middle-income countries (2–4). In Ethiopia, the absence of a standard registry and the prevalence of unsupervised deliveries hinder the understanding and management of congenital anomalies (5, 6).

Early detection through comprehensive newborn physical examinations is essential for improving developmental outcomes and guiding timely medical interventions (4). However, standardized examination protocols and routine screening are not widely practiced in Ethiopia, resulting in many anomalies remaining undiagnosed (5, 6). This study addresses a critical gap by exploring real-world newborn examination practices and congenital anomaly detection in major Ethiopian public hospitals, aiming to inform policy and improve early detection and management.

Research questions: (a) What are current newborn examination practices in large public hospitals in Addis Ababa? (b) What barriers and facilitators influence adoption of checklist-based examinations? (c) How are documentation and communication with parents managed?

## Methods

### Study design and setting

We employed a qualitative exploratory design across four public hospitals in Addis Ababa: Abebech Gobena Hospital, Zewuditu Memorial Hospital, Tirunesh Beijing Hospital, and Gandhi Memorial Hospital, collectively responsible for a substantial proportion of institutional deliveries in the city (7). The study period was from November 2024 to January 2025.

### Sampling strategy and participants

Purposive sampling was used to identify clinicians directly involved in neonatal care, including pediatricians, obstetrician-gynecologists, and midwives, each with at least two years of experience at their respective institutions. Twelve key informants participated (three per discipline across the four hospitals), ensuring representation of roles and sites. Sample size was justified by information power and thematic saturation.

### Data collection

Semi-structured interviews were conducted using a guide developed in English, translated into the local language, and back-translated to ensure clarity. Topics included examination workflows, use of checklists, documentation, training, and parent communication.

### Data analysis

Interviews were transcribed verbatim, translated into English, and analyzed in Open Code following Braun & Clarke's thematic analysis. Two researchers independently coded transcripts, met to resolve discrepancies, and iteratively refined a coding tree. Themes were validated through member checking and peer debriefing. Trustworthiness was supported by triangulation (roles and sites), an audit trail, and reflexive notes.

### Ethics

Ethical approval was granted by the Yekatit 12 Hospital Medical College Institutional Review Board (Ref. No: Y12HMC/729/09/24). Written informed consent was obtained from all participants. No minors were involved.

## Results

Twelve participants (pediatricians, obstetrician-gynecologists, and midwives) from four public hospitals in Addis Ababa contributed perspectives on examination practices, training, documentation, and institutional readiness. Hospitals reported between 400 and 1,100 deliveries per month (typically around 500). Results are organized into five thematic findings with illustrative quotations.

### Theme 1: Patient flow and delivery volumes

Most expectant mothers access the study institutions either through referrals from their catchment health centers or by presenting directly from the community via the emergency department. The majority of neonates are delivered at the hospitals themselves, with a smaller proportion referred from surrounding catchment areas. A gynecologist participating in the study explained:

“Mostly they come with a referral from the catchment areas. Sometimes we also accept self-referrals from the catchment health centers and those who have delivered at our center before…”

A pediatrician added:

“We receive the neonates delivered in our hospital either vaginally or via cesarean section (CS). Neonates are also referred from health centers in the catchment areas. Sometimes they come directly from the community through the emergency department without a referral.”

The respondents clearly indicate that the four institutions typically handle between 400 and 1,100 deliveries each month, with an overall average of 500 deliveries per month.

Laboring mothers are triaged before being sent to the labor ward. After delivery, mothers who have had a spontaneous vaginal delivery (SVD) are monitored in the postnatal ward for several hours. Healthy newborns remain with their mothers, while those with complications are transferred to the Neonatal Intensive Care Unit (NICU). Maternal vital signs are monitored, and neonates undergo a gross evaluation in the postnatal ward. A midwife illustrated unanimously:

“After delivery, mothers who had a spontaneous vaginal delivery go to the postnatal ward to be observed for six hours. Immediately after delivery, the neonate is placed on the mother's abdomen and delayed cord clamping is practiced, with a gross examination done at the same time. Essential newborn care is given under the radiant warmer. Well babies are kept by the mother's side, and any newborn with problems is sent to the NICU. At the postnatal ward, the mother's vital signs are followed, and the neonates are grossly evaluated.”

### Theme 2: Fragmented newborn examination process

The approach to newborn physical examination varies widely across institutions. While most facilities aim to examine newborns immediately after delivery, the structure and thoroughness of these examinations depend on institutional resources and staff availability. At one hospital, year-one gynecology residents are responsible for examining each newborn, regardless of delivery mode. A gynecologist also mentioned:

“After delivery, all neonates are examined by at least a year-one gynecology resident. If any problem is identified, senior residents further evaluate the neonate.”

In other institutions, neonates may be examined by neonatal nurses, midwives, or general practitioners.

A pediatrician highlighted a limitation:

“After delivery, mothers who had SVD are transferred to the postnatal ward for observation. Babies without obvious problems stay with their mothers, while those with issues are transferred to the NICU. In the postnatal ward, the mother's vital signs are checked, and neonates are grossly evaluated. Midwives and general practitioners are responsible for the postnatal ward, but most of the time the newborn tends to be forgotten and not looked after well by the obstetrics team.”

Pediatricians may attend deliveries in the operating room if a high-risk birth is anticipated. As one pediatrician noted:

“If we anticipate delivery of a neonate with serious problems, a pediatrician will be available during delivery.”

Routine examinations are often inconsistent, focusing on gross physical assessments without specialized follow-up unless an anomaly is readily visible. Another pediatrician remarked:

“It is very difficult to say that every newborn will be evaluated by pediatricians properly because visits are brief and done only once per day.”

Participants emphasized that obvious anomalies like hydrocephalus and limb defects are usually detected, but subtle conditions (e.g., cardiac murmurs, imperforate anus) commonly overlooked, leading to delayed treatment and adverse outcomes. A pediatrician explained:

“There are many congenital anomalies, but the ones detected are the gross ones like hydrocephalus, cleft lip and palate, limb abnormalities, and spinal defects. Subtle anomalies may not be detected early. For example, cardiac murmurs may go unnoticed unless examined properly. Imperforate anus is often missed. We had a neonate with imperforate anus who was sent home undetected, developed complications, and died. The anomaly was only detected after complications arose.”

Most participants reported that commonly identified anomalies are those obvious to both clinicians and parents, such as neural tube defects, hydrocephalus, Chiari II anomalies, cleft lip, and palate. Advanced ultrasound is used to detect major anomalies prenatally, such as spina bifida and hydrocephalus. A gynecologist stated:

“The majority of congenital anomalies detected are spina bifida and hydrocephalus. If a severe anomaly incompatible with life is detected by ultrasound, the parents are advised to terminate the pregnancy.”

Antenatal scans are routine for high-risk pregnancies at some institutions.

Participants noted that essential materials such as spatulas and torches for oral examination are not available in postnatal wards. As one midwife stated:

“Simply they observe, but there are no spatula and torch to examine the infant.”

A checklist-based structured physical examination is not routinely practiced before discharge. A midwife explained:

“General practitioners work in the postnatal ward, but they do not perform a checklist-based structured head-to-toe physical examination of the newborn before discharge unless there is a complaint. There is also a communication failure; some think the examination is done immediately after delivery.”

Practices regarding structured checklists for neonatal examination vary. Some institutions have recently developed checklists for congenital anomalies but have not yet implemented them. Others rely on routine assessments without structured tools. As a gynecologist noted:

“There's no checklist or standard method of examining the newborns; just a general gross examination is made immediately after delivery.” Another gynecologist added:

“There is a checklist developed recently targeted for birth anomalies but not used.”

### Theme 3: Inadequate training and supervision

Non-physician staff, such as midwives and neonatal nurses, are involved in examining, caring for, and discharging newborns, but often lack adequate training. Participants noted variability in the ability and skill of professionals due to insufficient training. A midwife explained:

“All mothers and newborns are evaluated, but there are personal differences in how well they are evaluated because of inadequate training and skill gaps among medical professionals.”

Nearly all participants emphasized the need for training delivery ward staff in standardized newborn physical examination, which would improve detection and timely management of less visible congenital anomalies.

Participants explained that efforts to improve neonatal care face challenges such as limited trained staff and resource shortages. A pediatrician explained:

“I initiated visits to the postnatal wards, but we could not maintain this due to staff shortages and poor interdepartmental communication, which affects timely patient evaluation. I want to continue this practice because it helps to identify not only congenital anomalies but also hypoglycemia and hypothermia, which can be fatal for neonates.”

### Theme 4: Documentation gaps and communication challenges

Documentation of newborn physical examinations and congenital abnormalities faces several challenges, including the lack of a standardized and structured approach. While electronic medical records (EMRs) are used, there are significant inconsistencies in documentation quality. Records are often incomplete, especially when newborns do not present with obvious concerns. A participant noted:

“The physical examination of a newborn is recorded in the EMR, but I'm not sure if it's routinely done. Also, I'm not sure if proper evaluation and presence or absence of congenital anomalies are documented on the charts at the postnatal units.”

In most institutions, documentation is inconsistent and often only completed when newborns require referral to the NICU. The EMR typically includes a structured “delivery summary” (APGAR score, time of delivery, weight, vitamin K administration, complications, interventions) but lacks a specific section for congenital anomalies.

Some participants explained that they faced different challenges when they communicate the congenital anomalies detected to parents or families. Some of the challenges were the parents shocked, unable to understand and refused to accept the anomalies identified. This can be evidenced by the response of one participant:

“Families are usually shocked and they may find it difficult to understand, especially if they have multiple limb abnormalities or significant facial features. Most mothers aren't ready to accept it after delivery because it's far from their expectation”

### Theme 5: Institutional support and willingness for checklist-based practices

Participants identified the adoption of checklist-based newborn physical examinations as a promising solution for improving anomaly detection. Several acknowledged the potential benefits of structured checklists in enhancing detection and management. One obstetrician/gynecologist stated:

“The checklist will be very useful to detect, record, and timely manage congenital anomalies.”

The study confirmed that while structured checklist-based examinations are lacking, healthcare providers are willing to adopt them. As participants explained:

“We believe our institutions will welcome a checklist-based screening; of course, there may be some challenges and resistance during the initial implementation, but most professionals will welcome it very well.”

All participants agreed that there is a gap in detecting congenital anomalies at their institutions, similar to other public hospitals. They emphasized the importance of implementing checklist-based structured examinations nationwide, while acknowledging challenges such as the need for trained and motivated professionals, and support from policymakers and medical staff.

## Discussion

This study highlights significant gaps in neonatal examination practices, training, and documentation in major Ethiopian public hospitals. The lack of standardized protocols and routine use of structured checklists compromises the quality and consistency of neonatal care, leading to missed diagnoses and delayed interventions.

The study institutions, being the largest delivery and teaching centers in the city, play a pivotal role in shaping neonatal care standards. The absence of a unified examination protocol in these settings compromises the safety, quality, and continuity of neonatal care.

International literature supports the importance of standardized newborn examinations conducted by trained professionals, with checklists serving as valuable tools for comprehensive assessment.

The newborn physical examination is a comprehensive assessment and a cornerstone of the Universal Child Health Promotion Program (8). Its timing should correspond with the physiological adaptations that neonates undergo as they transition to extra uterine life. This examination supplements the initial general assessment typically performed by the midwife or neonatal team immediately after birth to confirm the absence of obvious abnormalities (9).

Our study reveals significant inconsistencies in the implementation of standardized protocols for newborn physical examinations. While some institutions are in the process of developing checklists to guide newborn examinations, others continue to rely on unstructured general assessments. Such variability raises concern about the adequacy of care; as critical assessments may be overlooked. This finding indicate that without standardized protocols, the quality of neonatal care is at risk, potentially resulting in missed diagnoses of congenital anomalies and other health issues.

In contrast, countries like the United Kingdom have implemented standardized newborn and infant physical examinations nationwide, though operational details may vary by region (10). Davis and Elliman (10) recommend that newborn examinations be conducted by suitably trained and competent healthcare professionals with ongoing clinical experience. This standard is not consistently met in our study institutions, as echoed by nearly all participants. Regardless of a provider's background or qualifications, the quality and content of the examination should remain consistent across all facilities. Tappero and Honeyfield (11) further highlight that inadequate knowledge and skills among healthcare providers can lead to missed minor and subtle congenital anomalies a concern echoed by our participants, who noted that such anomalies are often overlooked due to insufficient training and the absence of structured, checklist-based examinations.

Our findings also highlight concerns regarding the training and competency of healthcare providers involved in newborn examinations. Many participants noted that non-physician staff, such as midwives and neonatal nurses, often lack the necessary training to conduct thorough assessments. Most professionals working with neonates have not received targeted training in anomaly detection, resulting in inconsistencies in care and a higher likelihood of missed diagnoses. This underscores the need for regular training programs to address gaps in knowledge and practical skills. Continuous education is essential for improving detection rates of subtle anomalies that might otherwise go unnoticed. Similar findings have been reported elsewhere; for example, Kaur et al. (12) emphasized the importance of on-the-job training and accessible clinical guidelines for nurses in neonatal care. Additionally, evidence suggests that providers' skills improve with adequate training, performing a minimum number of examinations annually, and attending relevant forums (9, 13).

The use of structured checklists for infant physical examinations varied significantly across the study sites. Most participants reported reliance on gross examinations without structured approaches, which may compromise thoroughness and accuracy. Standardizing checklist use across facilities could improve clinical practice by ensuring consistent and comprehensive evaluations, ultimately enhancing detection rates for congenital anomalies. This is supported by the Queensland Clinical Guidelines (14), which emphasize the importance of checklists in newborn examinations for systematically assessing body systems and detecting conditions such as heart defects and hip dysplasia. The value of such checklists as screening tools is further supported by Rogers et al. and Poojari et al. (15, 16).

Regarding congenital anomaly detection, while major anomalies such as hydrocephalus are often identified prenatally via advanced ultrasound, subtle conditions frequently go undetected until after birth. This inconsistency can delay interventions and worsen outcomes. Our findings align with literature indicating that operator dependency and limitations in ultrasound technology contribute to low detection rates for subtle anomalies during prenatal screening. Van Nisselrooij et al. (17) noted that perinatal diagnoses may be missed due to impaired clinical skills and lack of experience. Poojari et al. (15) recommend the development and implementation of standard protocols for prenatal screening across all obstetric care facilities, while Rogers et al. (16) reaffirm the value of structured checklists for newborn anomaly detection.

Participants in our study reported commonly identified anomalies such as neural tube defects, hydrocephalus, Chiari II anomalies, and cleft lip and palate. Major anomalies like spina bifida and hydrocephalus are typically detected prenatally, consistent with findings from Jimma Medical Center, which also reported myelomeningocele, hydrocephalus and anorectal anomalies as prevalent anomalies (18).

Documentation of newborn physical examinations and congenital abnormalities is primarily electronic, though some hospitals still use paper records. However, the lack of a standardized and structured documentation approach leads to inconsistencies and incomplete records. Even though this study did not quantify documentation completeness, our findings confirm that most public hospitals' records are inconsistent, a result echoed by studies in Benin-city and elsewhere, which found poor documentation of external genital examinations and identified incomplete records as a persistent challenge in neonatal care (15, 19).

Finally, participants described challenges in communicating congenital anomaly findings to parents, including parental shock, misunderstanding, and denial. This finding is supported by the qualitative study finding done in Jordan and Saudi Arabia which stated that mothers experienced shock when they heard about their child congenital anomalies for the first time (20). Participants noted that breakdowns in communication may further delay treatment initiation and worsen parental anxiety, highlighting the need for improved counseling frameworks and sensitivity training in neonatal care settings (21, 22). These challenges further highlight the need for standardized protocols, comprehensive training, and effective communication strategies to improve neonatal outcomes.

## Limitations

The study's qualitative design and focus on urban public hospitals may limit the generalizability of findings to other settings, including rural or private institutions. Social desirability bias and reliance on self-reported practices without direct observation or record audits may have influenced findings. Nevertheless, triangulation of roles and sites and member checking strengthened credibility.

## Conclusions

This study demonstrates that newborn physical examination practices at the assessed public health facilities in Addis Ababa are inconsistent and lack standardization. There is no established protocol specifying which health professionals should conduct newborn examinations, nor are there defined competency or training requirements. Documentation of examination findings is not routinely performed unless abnormalities are detected, and a comprehensive, checklist-based head-to-toe assessment prior to discharge is absent. Additionally, essential materials such as spatulas and torches for oral examinations are often unavailable in postnatal wards.

Major congenital anomalies, such as spina-bifida and hydrocephalus, are typically identified through prenatal ultrasound; however, subtle conditions frequently go undetected during both prenatal and postnatal assessments. Given that these institutions are the largest delivery and teaching hospitals in Addis Ababa, it is likely that these findings reflect practices in other public health facilities in Ethiopia.

## Recommendations

We recommend the development and implementation of a standardized national neonatal examination checklist to guide comprehensive newborn assessments; establish mandatory protocols specifying the roles, competencies, and training requirements for healthcare providers conducting newborn examinations; initiate comprehensive training programs for healthcare providers to enhance skills in conducting thorough newborn examination with a particular focus on early detection of congenital anomalies; and, support and conduct community-based studies on the incidence of birth defects to inform data-driven interventions and policy development.

## Data Availability

The raw data supporting the conclusions of this article will be made available by the authors, without undue reservation.
